# Supplementary material for: Improving the odds of survival: transgenerational effects of infections
Source: EMBO Mol Med. 2025 Jan 22;17(4):609–24. doi: 10.1038/s44321-025-00192-9 (PMC11982362; doi:10.1038/s44321-025-00192-9)
Supplement: Supplementary file 1 — Table EV1 [file 44321_2025_192_MOESM1_ESM.docx]

| Host Taxonomy  Phylum/Class/Order/Species | | Maternal vs Paternal vs Parental | Intragenerational vs Intergenerational vs Transgenerational | Infection/ Immune activation | Key Findings/Effects | Literature |
| --- | --- | --- | --- | --- | --- | --- |
| Arthropoda/Insecta | | | | | | |
| *Lepidoptera* | *Plodia interpuctella* (Indian meal moth) | Parental | Intergenerational | *Plodia interpunctella* granulosis virus | F0: reduced susceptibility to a subsequent lethal viral challenge.  F1: less susceptible to viral challenge. | ([Tidbury *et al.*, 2011](#_ENREF_104)) |
|  | *Galleria mellonella* (wax moth) | Parental | Transgenerational (up to 25 generations) | *Beauveria bassiana* pathogenic fungus | F1 to F25: enhanced resistance to *Beauveria bassiana.* | ([Dubovskiy *et al.*, 2013](#_ENREF_24)) |
|  | *Galleria mellonella* (wax moth) | Parental | Transgenerational (up to 20 generations) | *Bacillus thuringiensis* entomopathogenic bacterium | F5 to F20: resistance to *Bacillus thuringiensis.* | ([Dubovskiy *et al.*, 2016](#_ENREF_23)) |
|  | *Manduca sexta* (tobacco hornworm) | Parental | Intergenerational | Non-pathogenic *Escherichia coli* or *Serratia entomophila* | F1: sex specific TGIP. | ([Gegner *et al.*, 2019](#_ENREF_37)) |
| *Coleoptera* | *Triboleum castaneum* (red flour beetle) | Paternal | Intergenerational | Knockdown of *Dnmt2* via pupal RNAi + *Bacillus thuringiensis* entomopathogenic bacterium | F1: increased mortality after *Bacillus thuringiensis* infection indicating an increased stress sensitivity caused by the lack of *Dnmt2.* | ([Schulz *et al.*, 2022](#_ENREF_89)) |
|  | *Triboleum castaneum* (red flour beetle) | Paternal | Intergenerational | *Bacillus thuringiensis* entomopathogenic bacterium | F1: only the genetic offspring of immune primed males showed enhanced survival upon bacterial challenge while phenol oxidase activity was increased in both genetic and step offspring sired by different males. | ([Eggert *et al.*, 2014](#_ENREF_26)) |
|  | *Triboleum castaneum* (red flour beetle) | Paternal vs maternal | Intergenerational | Heat-killed or live *Bacillus thuringiensis* and *Escherichia coli* | F1: improved survival after challenge with live bacteria with the offspring exposed to the same bacteria as their parents survive longer. TGIP was consistent for maternal and paternal priming. | ([Roth *et al.*, 2010](#_ENREF_82)) |
|  | *Triboleum castaneum* (red flour beetle) | - | Intragenerational | Heat-killed and live *Escherichia coli*, *Bacillus thuringiensis* 1 (isolated from a Mediterranean flour moth), *Bacillus thuringiensis* 2 and *Bacillus subtilis* | F0: beetles exposed to previous immune priming with heat-killed bacteria survive significantly longer after a subsequent homologous challenge (the same bacteria twice) than heterologous. | ([Roth *et al.*, 2009](#_ENREF_83)) |
|  | *Tenebrio molitor* (mealworm beetle) | - | Intragenerational | Lipopolysaccharide (pre-challenge) and *Metarhizium anisopliae.* entomopathogenic fungus | F0: pre-challenged larvae exhibit a long-lasting antimicrobial response providing them a survival benefit to fungal infections. | ([Moret & Siva-Jothy, 2003](#_ENREF_68)) |
|  | *Tenebrio molitor* (mealworm beetle) | Parental | Intergenerational | Lipopolysaccharide | F1: parental challenge improved offspring immunity via the production of antimicrobial peptides in the hemolymph. | ([Moret, 2006](#_ENREF_66)) |
| *Hymenoptera* | *Apis mellifera (honeybee)* | Maternal (worker bees) | Transgenerational (colony) | Heat-killed: *Ascosphaera apis* spores (fungi) and *Paenibacillus* larvae cells (bacteria) | Young worker larvae: high resistance to *Paenibacillus* larvae infection and enhanced social immunity to pathogens through TGIP via the vitellogenin-hypopharyngeal gland axis. | ([Kim *et al.*, 2023](#_ENREF_50)) |
|  | *Apis mellifera (honeybee)* | Maternal (queen) | Transgenerational (colony) | *Melissococcus plutonius* | Offspring larvae: no evidence of TGIP, neither natural nor experimental exposure of the queens induced protection of larvae upon *Melissococus plutonius* infection. | ([Ory *et al*, 2022](#_ENREF_73)) |
|  | *Apis mellifera* subsp. *carnica (honeybee)* | Maternal (queen) | Transgenerational (colony) | Heat-killed *Paenibacillus larvae* (spore-forming bacterium) causative agent of American foulbrood | Offspring larvae: strong TGIP effect on offspring immunity enhancing their survival rate by 26.0% to American foulbrood infection. | ([Hernández López *et al*, 2014](#_ENREF_44)) |
|  | *Bombus terrestris (buff-tailed bumblebee)* | Maternal (queen) | Transgenerational (colony) | Lipopolysaccharide | Male offspring: higher phenol oxidase activity  Female offspring: higher antibacterial activity but lower phenol oxidase activity. | ([Moret & Schmid-Hempel, 2001](#_ENREF_67)) |
|  | *Bombus terrestris (buff-tailed bumblebee)* | Maternal (queen) | Transgenerational (colony) | Heat-killed bacteria *Arthrobacter globiformis* | Adult worker offspring: TGIP is mediated through factors in the eggs which increase their internal antibacterial activity with these effects persisting into the adult worker offspring. | ([Sadd & Schmid-Hempel, 2007](#_ENREF_88)) |
|  | *Bombus terrestris (buff-tailed bumblebee)* | Maternal (queen) | Transgenerational (colony) | Heat-killed bacteria *Arthrobacter globiformis* (queen), lipopolysaccharide (offspring) | Adult worker offspring: TGIP evidenced higher levels of antibacterial activity in the worker offspring of immune challenged mother queen. | ([Sadd *et al*, 2005](#_ENREF_87)) |
| *Diptera* | *Drosophila melanogaster (fruit fly)* | Parental | Transgenerational (F> 50) | Transgene insertion | F>50: epiallele inheritance via polycomb dependent chromatin marks: existence of robust TEI. | ([Ciabrelli *et al.*, 2017](#_ENREF_18)) |
|  | *Drosophila melanogaster (fruit fly)****,*** *Aedes aegypti (yellow fever mosquito)* | Maternal | Transgenerational (up to F5) | Different RNA viruses | F1-F5: antiviral TGIP with long lasting protection throughout generations which is virus and sequence specific. | ([Mondotte *et al*, 2020](#_ENREF_62)) |
|  | *Aedes aegypti (yellow fever mosquito)* | Maternal | Intergenerational | Immune priming with Dengue virus different serotypes | F1: lower susceptibility to dengue infection but trade-off in life history (females) including reduction of pupation time, negative effect on pupation percentage, shift in the sex ratio towards females. | ([Cime-Castillo *et al*, 2023](#_ENREF_19)) |
|  | *Culex pipiens* | Maternal | Intergenerational | *Plasmodium relictum* | F1: no evidence of transgenerational protection, fecundity loss. | ([Pigeault *et al.*, 2015](#_ENREF_77)) |
|  | *Anopheles coluzzii* *(Malaria vector mosquito)* | Parental | Intergenerational | *Chromobacterium anophelis* | F0: reduced reproduction capacity by reducing insemination rate.  F1 larvae: reduction of fitness, size, and survival. | ([Gnambani *et al*, 2023](#_ENREF_38)) |
|  | *Aedes aegypti (yellow fever mosquito)* | Maternal | Transgenerational (up to F3)  F4 and F5: upon reactivation | Alphavirus, Semliki Forest virus | F1-F3: specific protective immunity to homologous virus  F1: resistance to related heterologous alphavirus | ([Rodriguez-Andres *et al*, 2024](#_ENREF_81)) |
| *Arthropoda/Bronchipoda* | | | | | | |
| *Diplostraca* | *Daphnia magna* | Maternal | Intergenerational | *Pasteuria ramosa* pathogenic bacteria | F1: strain-specific acquired immunity. | ([Little *et al.*, 2003](#_ENREF_57)) |
| *Anostraca* | *Artemia franciscana* | Parental | Transgenerational (up to F3) | Live or dead *Vibrio parahaemolyticus* and *Vibrio campbellii* | F1 to F3: increased survival upon exposure to homologous or heterologous *Vibrio* infection with more pronounced protection against homologous *Vibrio* strains. | ([Roy *et al.*, 2022](#_ENREF_85)) |
|  | *Artemia franciscana* | Parental | Transgenerational (up to F3) | Phloroglucinol (a polyphenol derivative organic plant-derived compound) | F1 to F3: increased resistance against biotic stress i.e. bacteria (*Vibrio parahaemolyticus* and *Vibrio harveyi*) and abiotic stress i.e. lethal heat shock. | ([Roy *et al*, 2019](#_ENREF_86)) |
|  | *Artemia franciscana* | Parental | Transgenerational (up to F3) | *Vibrio campbellii* and *Vibrio* strain H6 (belonging to the *Harveyi* clade) | F1 to F3: increased resistance towards a homologous rather than to a heterologous bacterial strain. | ([Norouzitallab *et al*, 2016](#_ENREF_71)) |
| *Mollusca/ Bivalvia* | | | | | | |
| *Pectinida* | *Chlamys farreri or Azumapecten farreri (Farrer’s scallop)* | Maternal | Intergenerational | Heat-killed *Vibrio anguillarum* | F1 (larvae and eggs): significantly lower mortality after bacterial challenge in the offspring from the immune stimulated mother scallops. | ([Yue *et al*, 2013](#_ENREF_116)) |
| *Nematoda/ Chromadorea* | | | | | | |
| *Rhabditida* | *Caenorhabditis elegans* | Parental (XX hermaphrodites) | Transgenerational (up to F12 generation) | *Salmonella enterica* and *Escherichia coli* | F1 to F12: not significant change to overall survival, but suppression of gene expression and survival variability to subsequent generations. Repeating pattern of survival times over the 12 generations in the control lineage. | ([Wibisono & Sun, 2023](#_ENREF_111)) |
|  | *Caenorhabditis elegans* | Parental (XX hermaphrodites) | Transgenerational (up to F4) | Isolated small RNAs from *Pseudomonas aeruginosa* (ncRNA: P11) | F0 to F4: pathogen avoidance (behavioral defense response) via a ncRNA-dependent mechanism. | ([Kaletsky *et al.*, 2020](#_ENREF_48)) |
|  | *Caenorhabditis elegans* | Parental (XX hermaphrodites) | Transgenerational (up to F4) | Isolated small RNAs from *Pseudomonas vranovensis* (small RNA: Pv1) | F0 to F4: pathogen avoidance (behavioral defense response) to pathogenic *P. vravovensis* but also to beneficial *P. mendocina* mediated by small RNAs. | ([Sengupta *et al*, 2024](#_ENREF_90)) |
|  | *Caenorhabditis elegans* | Parental (XX hermaphrodites) | Transgenerational (up to F4) | *Pseudomonas fluorescens* (small RNA: Pfs1) | F0 to F4: pathogen avoidance (behavioral defense response) regulated by small RNAs. | ([Seto *et al.*, 2024](#_ENREF_92)) |
|  | *Caenorhabditis elegans* | Parental (*fog-2* males, wild type and *fog-2* hermaphrodites defective in spermatogenesis) | Transgenerational (up to F4) | *Pseudomonas aeruginosa* | F0 to F4: pathogen avoidance passes through male and female germline mediated via TGF-β signaling in sensory neurons and Piwi/Argonaute small RNA pathway. | ([Moore *et al.*, 2019](#_ENREF_65)) |
|  | *Caenorhabditis elegans* | Parental (XX hermaphrodites) | Transgenerational (up to F4) | RNAi treatment with small RNAs from *Pseudomonas aeruginosa, Escherichia coli* | F0 to F4: pathogen avoidance via the Cer1 retrotransposon. | ([Moore *et al.*, 2021](#_ENREF_64)) |
| *Chordata/Aves* | | | | | | |
| *Galliformes* | *Gallus gallus (chicken)* | Maternal and paternal | Transgenerational (more than 25 generations) | Marek disease (alpha herpesvirus) | > F25: altered immunity: susceptibility or resistance to the disease. | ([He *et al*, 2023](#_ENREF_43)) |
|  | *Gallus gallus (chicken)* | Maternal and paternal | Intergenerational | Dietary supplement comprised of vitamins and fermentation extracts of dried *Bacillus subtilis*, *Aspergillus niger* and *Aspergillus Oryzae.* | F1 chicks: protection against *Salmonella enteritidis* infection, reduction of colonization via systemic and intestinal robust and distinct cytokine and chemokine response. | ([Swaggerty *et al*, 2023](#_ENREF_99)) |
| *Chordata/Actinopteri* | | | | | | |
| *Cypriniformes* | *Danio rerio (zebrafish)* | - | Intragenerational | Training: non-lethal dose of *Shigella flexneri, BCG, β-glucan*  Challenge: different bacterial strains (*Pseudomonas, Shigella, Staphylococcus)* | Zebrafish larvae: trained immunity shown as enhanced antimicrobial capacity of trained neutrophils by *Shigella* induced epigenetic reprogramming that differs from BCG and β-glucan training. | ([Gomes *et al*, 2023](#_ENREF_39)) |
| *Cypriniformes* | *Ctenopharyngodon idella (grass carp)* | - | Intragenerational | Grass carp reovirus subtype II | Age-dependent susceptibility to grass carp reovirus infection regulated via variations in DNA methylation and gene expression patterns. | ([He *et al*, 2022](#_ENREF_42)) |
| [*Pleuronectiformes*](https://www.ncbi.nlm.nih.gov/Taxonomy/Browser/wwwtax.cgi?mode=Undef&id=8252&lvl=3&keep=1&srchmode=1&unlock) | *Cynoglossus semilaevis* (chinese tongue sole) | - | Intragenerational | *Vibrio harveyi* | Disease resistance of the selected *C. semilaevis* families mediated by differential methylation patterns of immune related genes. | ([Xiu *et al*, 2019](#_ENREF_112)) |
| *Chordata/Mammalia* | | | | | | |
| *Rodentia* | *Mus musculus* (C57BL/6J) | Paternal | Transgenerational (up to F2) | Poly I:C immune activation | F1: increased depression-like behavior, altered stress response, transcriptomic changes in hippocampus, and increased immune responsivity (males only).  F2: reduced early-life bodyweight and some modest changes in anxiety-like behavior (males only). | ([Kleeman *et al.*, 2024](#_ENREF_52)) |
|  | *Mus musculus* (C57BL/6) | Paternal | Transgenerational (up to F2) | *Toxoplasma gondii* protozoan | F1, F2: behavioral abnormalities in a sex-dependent manner.  Causation: Zygotic microinjection of isolated sperm small RNA recapitulates behavioral changes. | ([Tyebji *et al.*, 2020](#_ENREF_106)) |
|  | *Mus musculus* (C57BL/6) | Paternal | Intergenerational | Polymicrobial sepsis (induced by cecal ligation and puncture) | F1: body weight reduction, dampen systemic and pulmonary immune response (only male). | ([Bomans *et al.*, 2018](#_ENREF_9)) |
|  | *Mus musculus* (BALB/c) | Maternal | Intergenerational | *Nippostrongylus brasiliensis* nematode | F0: altered gut and breastmilk microbiota.  F1: altered gut microbiota and altered immunity (increased frequencies and numbers of activated CD4 T cells) in spleen. | ([Nyangahu *et al*, 2020](#_ENREF_72)) |
|  | *Mus musculus* (C57BL/6, BALB/c) | Maternal | Intergenerational | Human Influenza virus  Immune activation by Poly I:C | F1: influenza: abnormal behavioral responses including deficits in PPI, in exploratory behavior and social interaction. Increased PPI responses to antipsychotic and psychomimetic drugs.  F1: poly I:C: PPI deficits | ([Shi *et al.*, 2003](#_ENREF_94)) |
|  | *Mus musculus* (C57BL/6N) | Paternal | Transgenerational (up to F3) | Immune activation by Poly I:C (maternal) | F1: sensorimotor gating impairments, reduced sociability and increased cued fear expression.  F2, F3: reduced sociability, increased cued fear expression and behavioral despair (novel emerged phenotype). | ([Weber-Stadlbauer *et al.*, 2017](#_ENREF_110)) |
|  | *Mus musculus* (C57BL/6) | Paternal | Transgenerational (up to F2) | Sublethal systemic infection with *Candida albicans* or a zymosan challenge for trained immunity induction | F1, F2: enhanced responsiveness to endotoxin challenge, alongside improved protection against systemic heterologous *Escherichia coli* and *Listeria monocytogenes* infection. | ([Katzmarski *et al.*, 2021](#_ENREF_49)) |
|  | *Mus musculus* (C57BL/6) | Maternal | Intergenerational | Human Influenza virus | F1: increased nNOS expression in hippocampi of neonates born to mothers exposed to Human Influenza virus during the second trimester of pregnancy. | ([Fatemi *et al*, 1998](#_ENREF_29)) |
|  | *Mus musculus* (C57BL/6) | Maternal | Intergenerational | Human Influenza virus | F1: short-term and long-lasting effects on brain development including altered pyramidal and nonpyramidal cell density values, pyramidal cell atrophy and brain enlargement, associated with abnormal behavior in adult mice. | ([Fatemi *et al.*, 2002](#_ENREF_28)) |
|  | *Rattus norvegicus* (Sprague Dawley) | Maternal | Transgenerational (up to F5) | Lipopolysaccharide | F1 to F3: hypertension due to impaired ability of salt excretion.  F4, F5: salt-sensitive hypertension. | ([Cao *et al.*, 2022](#_ENREF_14)) |
|  | *Onychomys torridus and Onychomys Arenicola* (Grasshopper mice) | - | Intragenerational | bark scorpion venom | *Onychomys sp*. mice showed higher levels of venom resistance than that reported for non-resistant *Mus musculus*. The venom resistance displayed intra- and interspecific variability depended on bark scorpion sympatry and allopatry. | ([Rowe & Rowe, 2008](#_ENREF_84)) |
| Chordata/Mammalia | | | | | | |
| Primates | Human | Maternal | Intergenerational | Influenza virus | F1: 7-fold increase of risk of schizophrenia during exposure at the first trimester of pregnancy. | ([Brown *et al.*, 2004](#_ENREF_10)) |
|  | Human | Maternal | Intergenerational | *Toxoplasma gondii* | F1: aOR of schizophrenia/schizophrenia spectrum disorders for subjects with high maternal *Toxoplasma* IgG antibody titers of 2.61, 95% CI:1.00-6.82. | ([Brown *et al.*, 2005](#_ENREF_11)) |
|  | Human | Maternal | Intergenerational | Herpes simplex virus type 2 | F1: significant association between maternal antibodies and psychotic illness. | ([Buka *et al.*, 2001](#_ENREF_12)) |
|  | Human | Parental | Intergenerational | Influenza pandemic in Sweden 1918-19 | Women: educational attainment reduction by 3-4 months of schooling and probability of college attendance drop by 3-5 percentage points if their mothers potentially experienced the Spanish flu as a fetal insult.  Men: educational attainment reduction by 4-7 months of schooling and probability of college attendance drop by 7-11 percentage points if their fathers were potentially prenatally exposed.  No mother to son nor father to daughter transmission of the health shock. | ([Richter & Robling, 2013](#_ENREF_79)) |
|  | Human | Maternal | Intergenerational | Influenza epidemic 1957 Helsinki | F1: increased risk for schizophrenia. | ([Mednick *et al*, 1988](#_ENREF_60)) |
|  | Human | Maternal | Intergenerational | SARS-CoV-2 | F1: Maternal SARS-CoV-2 positivity during pregnancy was associated with greater rate of neurodevelopmental diagnoses (OR 2.17, 95% CI: 1.24-3.79, *P* = 0.006). | ([Edlow *et al.*, 2022](#_ENREF_25)) |
|  | Human | Maternal | Intergenerational | Influenza pandemic 1918-19 | F1: Individuals exposed i*n utero* have altered family formation patterns (small overall effects on men but moderate effects for women). | ([Fletcher, 2018](#_ENREF_32)) |
|  | Human | Maternal | Intergenerational | SARS-CoV-2 | F1: SARS-CoV-2 *in utero* differentially alters methylation of genes in pathways that play a role in human neurodevelopment. | ([Hill *et al.*, 2023](#_ENREF_45)) |
|  | Humans  Respiratory Health in Northern Europe (RHINE) study | Parental | Intergenerational | *Mycobacterium tuberculosis* | F1: higher risk of asthma (aOR 1.29, 95% CI: 1.05-1.57) in offspring with a history of parental Tuberculosis (TB) as compared to offspring without parental TB.  Parental TB was significantly associated with allergic asthma in offspring. | ([Gyawali *et al.*, 2023](#_ENREF_40)) |
|  | Humans | Parental | Intergenerational | *Mycobacterium tuberculosis* | F1: asthma risk was higher in individuals with parental TB in childhood (OR 1.73, 95% CI: 1.20-2.50) or later preconception (OR 1.38, 95% CI: 1.00-1.91) than in the ones with parental TB after offspring´s birth; this was significant only in the maternal line (childhood: OR 1.95, 95% CI: 1.13-3.37; later preconception: OR 1.74, 95% CI: 1.08-2.80). | ([López-Cervantes *et al*, 2022](#_ENREF_59)) |

**Table EV1: A summary table of the literature and the key findings according to the host taxonomy (phylum/class/order/species)**

**Abbreviations:** adjusted Odds Ratio (aOR), Bacillus Calmette-Guerin (BCG), Confidence Interval (CI), DNA methyltransferase 2 (Dnmt2), neuronal Nitric Oxidase Synthase (nNOS), non-coding RNA (ncRNA), Odds Ratio (OR), Polyinosinic: Polycytidylic acid (Poly I:C), prepulse inhibition (PPI), RNA interference (RNAi), Tuberculosis (TB), Transgenerational Epigenetic Inheritance (TEI), Transgenerational Immune Priming (TGIP), Transforming Growth Factor-β (TGF-β).
